# Advances in Fluorescent Adjuncts in Pediatric Surgery: A Comprehensive Review of Applications of Indocyanine Green Across Surgical Specialties

**DOI:** 10.3390/children12081048

**Published:** 2025-08-09

**Authors:** Nicholas Jose Iglesias, Andres Mauricio Corona, Akshat Sanan, Eduardo Alfonso Perez, Carlos Theodore Huerta

**Affiliations:** DeWitt Daughtry Family Department of Surgery, Division of Pediatric Surgery, University of Miami Miller School of Medicine, Miami, FL 33136, USA

**Keywords:** pediatric surgery, fluorescence, indocyanine green, minimally invasive, surgical oncology

## Abstract

**Highlights:**

**What are the main findings?**

• While Indocyanine Green (ICG) is often associated with fluorescence of hepatobiliary pathologies and anatomy, its route and timing of administration can be titrated to target a wide variety of pathologies.
• ICG fluorescence can aid in assessment of vascular anatomy, biliary anatomy, congenital lesions, tumor margins, tissue perfusion, and lymphatic flow.

**What is the implication of the main finding?**

• ICG is a low-risk, low-cost adjunct that can aid in the surgical approach to numerous pathologies.
• Preoperative planning is required to adequately target pathology of interest with proper route of administration, ICG dosage, and timing of administration.

**Abstract:**

**Introduction:** Indocyanine green (ICG) dye is used in a myriad of medical and surgical applications and complications related to its use are exceedingly rare. ICG fluorescence can be detected in unique locations depending on route, dosage, and timing. Although ICG fluorescence is used more commonly in the adult population, its adoption in pediatric surgery has been increasing more frequently. This comprehensive review aims to elucidate the myriad of ICG surgical applications within the pediatric population and important clinical considerations for administration. **Methods:** PubMed was queried for pediatric surgical applications of indocyanine green. Surgical application, route of administration, dosage, ICG-related complications, and surgical impact of ICG fluorescence were analyzed. **Results:** In the pediatric population, ICG is used in a multitude of hepatobiliary, gastrointestinal, cardiothoracic, lymphatic, urologic, gynecologic, plastic, ENT, ophthalmologic, and neurosurgical procedures. Applications range from oncologic resections to benign and congenital reconstructions. Administration can be intravenous, intralesional, subcutaneous, inhaled, or enteric. Timing, dosage, and route of administration are dependent on the pathology of interest. **Conclusions:** ICG is a safe and useful adjunct for a wide variety of pediatric surgical applications. This comprehensive review aims to highlight administration considerations and the efficacy of ICG fluorescence in various surgical subspecialty pathologies. Future studies should continue to focus on how to integrate pathology-specific ICG fluorescence into intraoperative decision-making.

## 1. Introduction

Indocyanine green (ICG) was initially developed as a near-infrared photography dye by Kodak in the Second World War, but was ultimately approved by the United States Food and Drug Administration (FDA) in 1959 for human biomedical applications [1,2]. ICG is a fluorescent tricarbocyanine dye with peak spectral absorption at 790 nm [3]. After an ICG avid target is illuminated with an excitatory light source (750–800 nm), it can be imaged at longer, near-infrared (NIR), emission wavelengths (>800 nm) to identify pattern of ICG fluorescence [4]. Its amphiphilic profile permits rapid binding to circulating plasma proteins, namely α-1 lipoprotein, that are excreted almost exclusively into the biliary tract over the course of 24–48 h [4,5,6,7]. After injection, only 2% of the injected ICG is unbound and is rapidly transported into the biliary tract by glutathione S-transferase [8]. While initially used for hepatic, cardiac, and renal evaluation, its applications in medical and surgical fields alike have blossomed over the past 65 years [2,5,9]. The widespread dissemination of NIR capable laparoscopic and robotic camera equipment has made ICG use within the operating room easier than ever, and further clinical applications of ICG continue to be explored [10,11]. NIR capable towers are estimated to be equivalent in price when compared to non-NIR towers, and NIR towers are likely already integrated within major medical centers [12,13]. ICG dye itself is also relatively inexpensive, costing hospitals as little as $25–$50 per vial [12,13]. Within pediatric surgery, ICG is commonly used during benign and malignant hepatobiliary cases and in the assessment of bowel/tissue perfusion but additional use cases including the assessment of lymphatic drainage are reported. Applications of ICG within pediatric surgical fields also continue to expand, and some call for its use to be the standard of care in numerous surgeries [7,14]. Within pediatric surgical specialties, ICG remains in its infancy relative to adult surgical specialties, though adoption is increasing nationwide [7]. There remains a paucity of standardization of ICG techniques within pediatric surgery, necessitating further investigation [15]. This comprehensive review aims to elucidate the myriad of ICG surgical applications within the pediatric population and important clinical considerations for administration.

## 2. Materials and Methods

Manuscripts published between 1991 and 1 June 2025 were identified in the National Institute of Health National Library of Medicine PubMed and MEDLINE databases. Manuscript search criteria included “indocyanine green” OR “ICG” AND “Pediatric Surgery”. Manuscripts selected for further review included those describing surgery on patients less than or equal to 18 years old and the use of indocyanine green for preoperative planning, intraoperative guidance, or postoperative diagnostics. Manuscripts were excluded if the patient population was greater than 18 years old, used an alternative fluorescent dye, or if the full manuscript was not available in English (Table 1) Surgical application, route of administration, dosage, ICG-related complications, and surgical impact of ICG fluorescence were analyzed.

## 3. Administration Considerations

Due to the amphiphilic molecular structure and rapid plasma protein adherence, ICG fluorescence occurs in unique locations depending on route, dosage, and timing. Additionally, fluorescent penetration is approximately 5–10 mm, so appropriate preoperative planning for the pathology of interest is required to achieve a satisfactory signal-to-noise ratio [16,17,18]. Deeper lesions (e.g. 12 mm) have the capacity to go undetected and should be considered preoperatively [19]. There remains significant practice variability in dosage and timing of ICG injection; however, the FDA currently recommends a maximum daily dose of 2 mg/kg for pediatric patients [17,20]. Table 2 summarizes dosage and timing considerations when administering ICG intravenously for a wide variety of pathologies. Table 3 and Table 4 summarize dosage and timing considerations when administering ICG intralesionally or subcutaneously, respectively. Finally, Table 5 summarizes various miscellaneous routes of administration, including inhaled and enteric, along with their respective indications.

ICG’s widespread use in the adult population over the past 6 decades has verified ICG’s safety, with an allergy rate as low as ~1/42,000 and only rare case reports of anaphylaxis related to ICG administration [21,22,23]. Reactions are suspected to be related to the iodine used to stabilize the compound [24]. Within animal models, LD50 is as high as 60–80 mg/kg, orders of magnitude above what is currently administered in pediatric and adult populations [4]. Rarely, skin discoloration may occur, but this phenomenon is transient and relatively benign [25,26]. One study of ICG within 1270 pediatric surgery cases was completely free of ICG-related adverse events [7]. A consideration for anesthesiologists intraoperatively is ICG’s impact on pulse oximetry. ICG is associated with a transient decrease (<10%) in SpO2; however, this is self-limited and does not appear to have physiological consequences. The magnitude and duration of the SpO2 decrease appear linearly correlated with the ICG dose. However, this self-resolves within minutes of administration, even at high doses [27,28]. If surgeons request anesthesia colleagues to administer ICG intraoperatively, this transient impact should be communicated to reduce unnecessary interventions.

**Table 2:** Intravenous Administration of ICG in Pediatric Surgery—Timing and Dosage Considerations by Target.

Target	Timing	Most Common Dosage	Outliers	Considerations	References
Angiography	Intraoperatively	0.3–0.5 mg/kg	Doses as low as 0.02 mg/kg for renal microvasculature in transplant surgery. Others administer standardized doses of 2.5–5 mg rather than weight-based dosing.	Fluorescence is visible within 1 min. ICG has a 3 min half-life intravascularly.	[7,11,17,26,29,30,31,32,33,34,35,36,37,38,39,40,41,42,43,44,45,46]
Cholangiography	3–7 h Preoperatively	0.4–0.5 mg/kg	Doses as low as 0.1 mg/kg for infants and small children. Others administer a standardized dose of 2.5 mg for older children rather than weight-based dosing. Some administer ICG only 15 min preoperatively, whereas others administer up to 18 h preoperatively.	A shorter duration between administration and operation may result in a poor signal-to-noise ratio due to diffuse hepatic uptake.	[11,17,38,44,47,48,49,50,51]
Biliary Atresia	24 h Preoperatively	0.1 or 0.5 mg/kg	0.05 mg/kg at 24 h preoperatively, 1 h preoperatively, or both.	ICG can be used as an adjunct for the diagnosis of biliary atresia and intraoperative guidance.	[17,38,52,53,54,55]
Choledochal Cyst	4–8 h Preoperatively	0.1 or 0.5 mg/kg	-	Preoperative dosing reduces background noise from the hepatic parenchyma.	[17,56,57]
Primary Hepatic Malignancy	48–96 h Preoperatively	0.5 mg/kg	ICG dosage ranged from 0.2 to 1.5 mg/kg, with timing ranging from 24 to 138 h preoperatively.	A shorter duration between administration and operation may result in a poor signal-to-noise ratio due to diffuse hepatic uptake.	[11,17,18,19,20,38,50,54,58,59,60,61,62,63,64,65,66]
Pulmonary Metastases	24 h Preoperatively	0.5 mg/kg	ICG dosage ranged from 0.2 to 1.5 mg/kg, with timing ranging from 0 to 96 h preoperatively. Some administered doses as high as 4.5 mg/kg for metastases from non-hepatic primaries.	-	[11,19,20,31,38,54,58,59,61,67,68,69,70,71,72,73,74,75,76,77,78]
Peritoneal Metastases from Hepatic Primary	72 h Preoperatively	0.5 mg/kg	-	-	[38,79]
Congenital Lung Malformations	Intraoperatively	0.3 mg/kg	Dosage ranged from 0.1 mg/kg to 0.6 mg/kg.	-	[80,81,82,83]
Esophageal perfusion	Intraoperatively	0.1 mg/kg	The standard angiography dose of 0.5 mg/kg has been successfully used as well.	-	[26,36,50,73,84,85,86,87]
Colorectal	Intraoperatively	0.2–0.5 mg/kg	Doses as low as 0.01 mg/kg have been successfully used. Others administer a standard dose of 2.5 mg and will redose if there is inadequate fluorescence.	-	[31,34,88,89,90,91,92,93]
Pancreatic Lesions	Intraoperatively	0.5 mg/kg or 2 mg/kg	-	Variable success rates with nodule fluorescence.	[50,71]
Splenic Cysts	Intraoperatively	0.2–0.6 mg/kg	-	-	[94,95,96]
Renal Malignancies	24 h Preoperatively	1.5 mg/kg	Dosage ranges from 0.05 to 0.67 mg/kg.	If administered intraoperatively, malignancy may be hypo-fluorescent relative to healthy parenchyma.	[97,98]
Benign Renal Pathologies (Cyst, ureteral duplication, etc.)	Intraoperatively	0.2–0.3 mg/kg	-	-	[14,38,99,100]
Sarcoma	24 h Preoperatively	4–5 mg/kg	-	Decreased tumor fluorescence at standard dosing.	[11]
Retroperitoneal Malignancies	Intraoperatively	0.3 mg/kg	-	-	[101]
ENT Malignancies	24 h Preoperatively	1.5 mg/kg	-	-	[102]

**Table 3:** Intralesional Administration of ICG in Pediatric Surgery—Timing and Dosage Considerations by Target.

Target	Timing	Most Common Dosage	Outliers	Considerations	References
Soft Tissue Malignancies (Melanoma, Sarcoma, Squamous Cell Carcinoma) Lymph Node Targeting	30 min Preoperatively or Intraoperatively	4–5 mg injected in total across multiple quadrants	0.25 mg injected in 4 quadrants 4–24 h preoperatively.	Lymphatic vessels and nodes are fluorescent.	[103,104,105,106]
Nephroblastoma Lymph Node Targeting	Intraoperatively	5 mg injected into the renal parenchyma or perihilar region.	-	Sentinel nodes are fluorescent.	[97,98,107]
Para-testicular Rhabdomyosarcoma Lymph Node Targeting	Intraoperatively	10 mg injected within the spermatic cord.	-	Sentinel nodes are fluorescent.	[17,108]
Pulmonary metastases/nodules	Immediately Preoperatively	0.0125–0.0625 mg injected into the target lesions under CT guidance.	-	Smaller lesions may require smaller volume injections.	[11,109,110]
Benign Cervico-facial Cysts (Thyroglossal, nasal, etc.)	Immediately Preoperatively	0.5–5 mg	-	Dosage depending on cyst size	[111]

**Table 4:** Subcutaneous Administration of ICG for Lymphangiography in Pediatric Surgery—Timing and Dosage Considerations by Target.

Target	Timing	Most Common Dosage	Outliers	Considerations	References
Congenital ascites or chylothorax	Intraoperatively	0.1–0.25 mg injected into the first interdigital regions of hands and feet.	-	Fluorescence pattern directs interventional approach.	[25,112,113]
Postoperative Chylothorax	1 h Preoperatively	0.25 mg injected into the interweb spaces between the first and second toes.	Doses vary from 0.025 mg/extremity to 5 mg in the bilateral inguinal regions.	Identify fluorescent extravasation from the thoracic duct or another injured lymphatic vessel.	[11,17,38,114,115,116,117,118,119]
Primary/Congenital Lymphedema	Intraoperatively	1 mg injected into the first and fourth interweb spaces of each hand and foot.	Doses vary from 0.0125 mg/extremity to 2.5 mg/extremity.	-	[38,112,120,121,122,123]
Lymphatic Malformations	Intraoperatively	0.75 mg injected into the interdigital space of the ipsilateral extremity or 0.005–0.125 mg into multiple loci around the lymphatic malformation.	-	The fluorescence pattern may be used to quantify the severity of malformation.	[38,123,124,125,126]

**Table 5:** Miscellaneous Routes of Administration of ICG in Pediatric Surgery—Timing and Dosage Considerations by Target.

Route	Target	Timing	Most Common Dosage	Outliers	Considerations	References
Enteral	Duodenal Atresia	Intraoperatively	5 mL of 2.5 mg/mL ICG	-	Visualize the duodenal web through the intestinal wall.	[50,127]
Inhaled	Pulmonary Sequestration	30–60 min Preoperatively	0.25 mg/kg or 0.5 mg/kg	-	The region of sequestration does not fluoresce.	[24,128,129]
Intra-testicular	Varicocele	Intraoperatively	2.5–6.25 mg	-	Laparoscopic Palomo technique is the most common approach.	[14,34,38,48,99,130]
Intra-ureter	Non-functional renal moiety	Intraoperatively	10 mL 2.5 mg/mL ICG solution	-	-	[14]

## 4. Clinical Applications

### 4.1. Hepatobiliary

#### 4.1.1. Benign Gallbladder Pathologies

**Figure 1:**
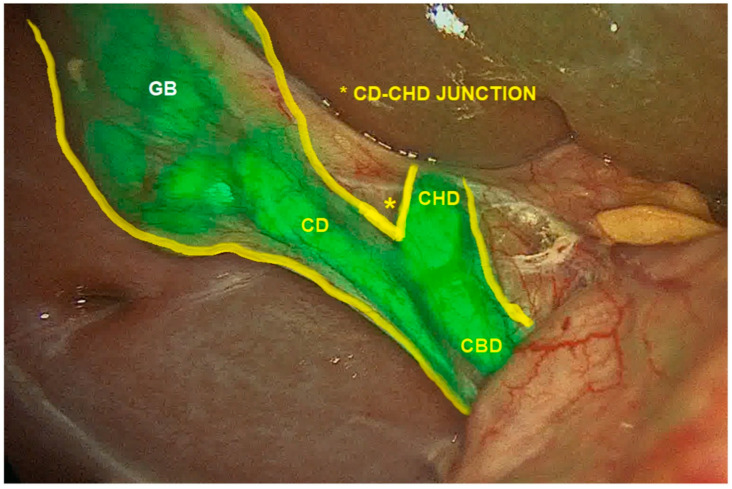
ICG Cholangiography identifying cystic duct, common bile duct, common hepatic duct, and cystic duct–common hepatic duct junction. *CBD*, common bile duct; *CD*, cystic duct; *CHD*, common hepatic duct; *GB*, gallbladder. Reprinted from C. Esposito et al. Indocyanine green fluorescent cholangiography: The new standard practice to perform laparoscopic cholecystectomy in pediatric patients. A comparative study with conventional laparoscopic technique. *Surgery* **2024**, *175*, 498–504, with permission from Elsevier [13].

**Figure 2:**
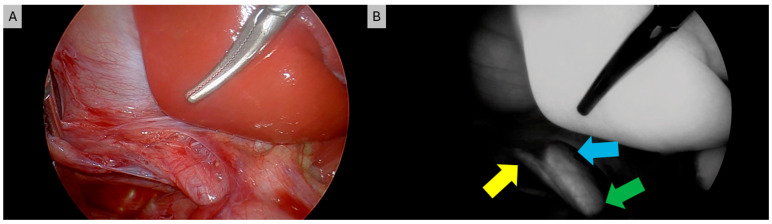
Laparoscopic cholecystectomy with (**A**) white light and (**B**) ICG cholangiography. Yellow arrow denotes short cystic duct initially not appreciated under white light alone. Blue arrow denotes common hepatic duct and green arrow denotes common bile duct.

As an exceedingly common surgery performed in pediatric and adult populations alike, laparoscopic and robotic cholecystectomy can be significantly augmented with the use of ICG cholangiography [7]. ICG cholangiography has been shown to aid in the delineation of biliary anatomy during the dissection of Calot’s triangle (Figure 1). Several studies have demonstrated augmented efficiency and visualization of the cystic duct–CBD junction in up to 100% of cases while also aiding in the identification of aberrant anatomy with the use of ICG cholangiography [13,16,31,38,44,131,132]. Figure 2 depicts one such case where a small cystic duct was initially not appreciated, but upon ICG fluorescence the cystic duct–CBD junction could be easily identified. In cases of complex congenital malformations, ICG may facilitate a safer dissection and faster visualization of the critical view of safety [13,38,47,49,131,132]. Furthermore, ICG cholangiography has reduced biliary manipulation and risk of ductal injury [12,133]. The use of ICG cholangiography is associated with a reduced incidence of open conversion due to this improved visualization of critical structures in some case series [13]. In the adult population, ICG use is associated with a reduced lifetime cost of >$1200 ($USD) per patient due to reduced operative time and reduced rates of conversion to open surgery [134]. Value analysis of ICG cholangiography suggests it may be more rapid, economic, and convenient compared to traditional fluoroscopic intraoperative cholangiography (IOC) [131,135]. Contrary to iodine-based IOC, ICG cholangiography does not require radiation or intraductal injection of contrast. Of note, ICG does not adequately visualize choledocholithiasis. If clinical suspicion is high, then traditional IOC should be obtained. Based on the improved visualization of the biliary tree anatomy and variations throughout the case with minimal risk, routine cholangiography with ICG has been recommended as standard of care for minimally invasive cholecystectomy approaches in the pediatric population [13].

#### 4.1.2. Biliary Malformations (Biliary Atresia and Choledochal Cysts)

While intraoperative cholangiography is the current gold standard for delineation of biliary anatomy in cases of biliary atresia, ICG is increasingly used to visualize biliary anatomy during hepatoportoenterostomy (Kasai procedure). ICG fluorescence has previously been utilized in confirming the diagnosis of biliary atresia and identifying the hepatic duct prior to dissection of the hyper-vascular neonatal liver [16,136,137]. Intraoperative ICG cholangiography is highly specific for the diagnosis of biliary atresia, as high as 100%, but the diagnosis cannot be ruled out with ICG cholangiography alone as sensitivity is as low as 64% [137]. Preoperatively, ICG fluorescence can be used as an adjunct in the diagnosis of biliary atresia by imaging fluorescence present in stool after injection, with only 1/12 falsely positive fluorescence, which the authors posit is due to progressive ductal fibro-obliteration [55]. ICG fluorescence of neonatal stool has a 97% accuracy for assessment of biliary patency [55].

Intraoperatively, the ICG fluorescence pattern after dissection of the fibrous cone assists in determining incision/anastomotic lines and may even predict clinical outcomes after Kasai procedure [52]. ICG fluorescence was noted in approximately 80% of hilar micro-bile ducts, and its use was even associated with improvement in bilirubin normalization postoperatively [53]. After biliary reconstruction, the flow of ICG into the alimentary tract can be visualized as well, confirming adequate reconstruction. Perfusion to the anastomosis can be assessed if ICG angiography is also performed [16,136]. Historically, ICG has been used to determine hepatic function after Kasai procedure by measuring plasma clearance rate after IV injection, although this technique has not gained widespread traction among surgeons [138].

Beyond biliary atresia, ICG cholangiography has aided in resection and reconstruction of choledochal cysts. The ICG fluorescence permits improved visualization of cyst dimensions and margins of healthy bile ducts intraoperatively, directing resection lines. ICG cholangiography may be performed via preoperative IV injection or by intra-biliary injection of ICG as described in Table 1 [139]. After reconstruction, ICG fluorescence can be visualized passing through the biliary reconstruction, verifying anastomotic patency and integrity [17,56,57,73].

#### 4.1.3. Hepatic Malignancies

**Figure 3:**
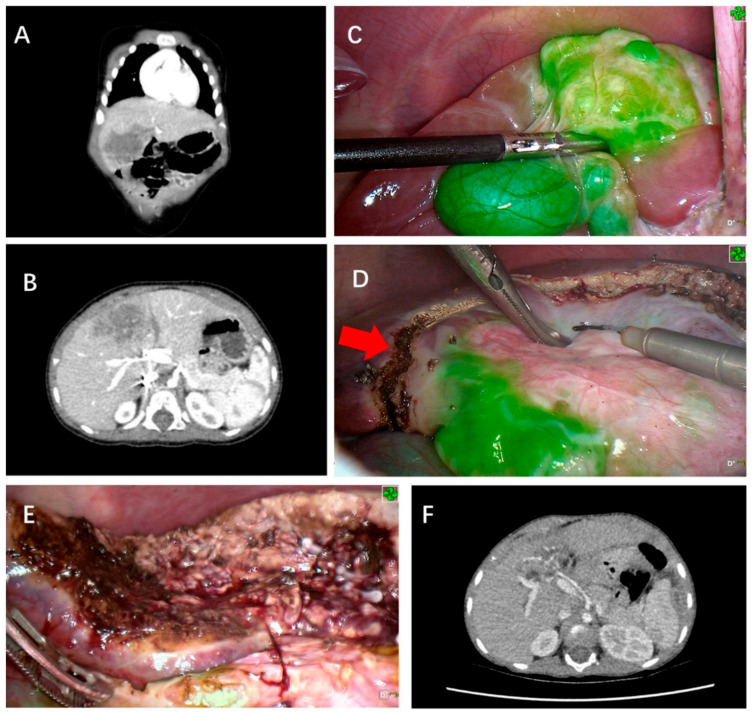
ICG fluorescence-guided laparoscopic hepatectomy of Segment 4a (**A**) Preoperative coronary CT examination of tumor. (**B**) Preoperative cross-sectional CT examination to measure the size of the mass. (**C**) Green fluorescence was observed in the S4 segment of the liver and gallbladder after the fluorescence mode was turned on. (**D**) Before liver removal, a marker line (red arrow) was made on the surface of the liver based on fluorescence imaging (**E**). After complete removal of the tumor, the incision margin was checked and no fluorescence was observed, indicating the complete removal of the tumor without bile leakage. (**F**) No signs of tumor residues or recurrence were observed via CT 12 months after surgery. Reprinted from R. Qiu et al. Deploying Indocyanine Green Fluorescence-Guided Navigation System in Precise Laparoscopic Resection of Pediatric Hepatoblastoma. *Cancers (Basel)* **2022**, *14*. Figure is licensed under Creative Commons-BY License [66].

The exclusive biliary excretion of ICG facilitates improved anatomic visualization of hepatic pathologies. ICG’s excretion from pathologic hepatoblastoma cells is delayed in relation to healthy hepatocytes, possibly due to microscopic biliary damage or mass effect from the malignancy [16,70]. This delayed excretion facilitates isolated hepatoblastoma fluorescence if IV ICG is administered preoperatively [140]. When ICG is administered 24–96 h preoperatively, intraoperative ICG fluorescence can aid in highlighting anatomic borders of the tumor and further guide the borders of resection (Figure 3) [11,19,31,38,58,59,60,62,63,64,73,141,142]. The fluorescence pattern consists of complete, partial, or only rim fluorescence [70,142,143]. Even following resection, ICG fluorescence can be visualized through the cut edge, offering augmented intraoperative visualization of pathologic margins [144]. This is particularly useful for small tumors that may not be palpable or visible intraoperatively [59,62,142,145]. In one case of a ruptured hepatoblastoma with peritoneal dissemination, ICG fluorescence guided metastasectomy of numerous fluorescent lesions with significant reduction in disease burden [79]. ICG fluorescence has even helped identify pathologically confirmed hepatoblastoma in nodules not initially detected on preoperative imaging in several studies [18,50,60]. Pathologic margins after ICG-guided partial hepatectomy for hepatoblastoma are congruent with visual interpretation of ICG fluorescence in 90–100% of cases [60,66,140,141]. Surgeons may encounter falsely positive fluorescent nodules that are later revealed to be pathologically negative, but this is infrequent [73].

Due to the limited penetrance of ICG fluorescence (<1 cm), deeper tumors may not be visualized, necessitating adjuncts such as intraoperative ultrasound for adequate localization and resection [19,145]. While hepatoblastoma is characteristically ICG fluorescent, other hepatic tumors, such as undifferentiated embryonal sarcoma of the liver, may not demonstrate ICG uptake by the tumor. In such cases where the malignancy is not fluorescent, a rim of fluorescence at the border of healthy hepatocytes may be present [143]. In patients with hepatic dysfunction, dysplasia, vascular irregularities, or cirrhosis, ICG’s delayed clearance from the patient’s dysfunctional hepatocytes may result in false-positive fluorescence [70,145,146]. Additionally, tumors with significant histologic necrosis after neoadjuvant chemotherapy, those that demonstrate teratoid features, or are metastases from non-hepatic primaries may have reduced or be non-fluorescent due to the reduced ICG uptake by these tissues [60,66,73]. An alternative technique for ICG-guided partial hepatectomy is by ligating the vascular supply to the intended segment for resection, then performing ICG angiography, which can delineate the border of de-vascularized parenchyma for resection [64]. Finally, ICG cholangiography can be utilized to identify bile leakage after partial hepatectomy, guiding ligation of small transected ducts along the cut edge [50,147,148].

#### 4.1.4. Transplantation

ICG angiography is increasingly utilized within the field of liver transplantation to guide anatomic resections via intra-operative ICG angiography during procurement and transplantation [149]. Even in patients pre-transplantation requiring spleno-renal bypass, ICG angiography is used to verify anastomotic patency intraoperatively [29]. During transplantation, ICG angiography aids in the assessment of graft perfusion beyond intraoperative Doppler. Furthermore, ICG angiography and cholangiography are useful in the management of post-transplantation complications such as hepatic arterial thrombosis and bile leaks, guiding surgical management [149,150].

### 4.2. Gastrointestinal Perfusion

#### 4.2.1. Esophageal

The esophagus is notoriously prone to ischemic injury and subsequent complications such as strictures or leaks inherently related to its tenuous vascular supply, especially after major reconstruction for pathologies such as esophageal atresia [151]. ICG angiography may serve as a novel component in the pediatric surgeon’s armamentarium in assessing the vascular supply to this delicate organ during open and minimally invasive approaches [87]. In cases of long gap esophageal atresia requiring initial internal traction, ICG angiography can aid in the verification of perfusion to the proximal and distal noncontiguous ends of the esophagus and the final anastomosis during reconstruction [36]. Some groups are currently using the ICG fluorescence pattern at esophageal anastomotic sites as part of a comprehensive esophageal anastomotic scoring system to stratify patient risk for postoperative anastomotic complications [84]. In cases of trachea-esophageal fistulae with recurrence, ICG angiography has been used to verify and preserve adequate perfusion to the esophagus during re-operation in hostile, adhered fields [26,85]. Similarly, in cases of redo Nissen fundoplication, perfusion of the distal esophagus may be difficult to visualize and preserve in a densely adhered field. ICG angiography provides visualization of key vessels by fluorescing through the adhesions, which may help facilitate safer dissection in re-operative fields [50,86]. In patients with long-segment destructive esophageal pathologies (e.g., caustic ingestion), reconstruction with colon or small bowel may be required. ICG angiography has been utilized within the pediatric population to visualize the vascular pedicle to the segment of bowel used for reconstruction and verify perfusion to the conduit and anastomosis [73].

#### 4.2.2. Small Bowel

**Figure 4:**
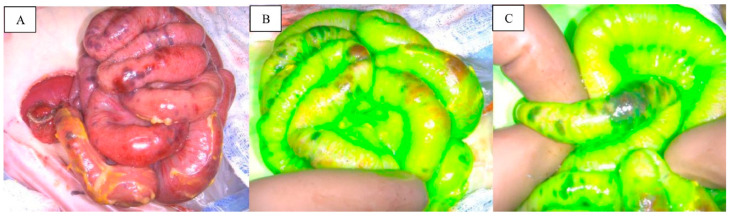
ICG angiography of necrotizing enterocolitis. Heterogeneous areas of possible ischemia or hematoma of the bowel wall under normal light (**A**) and fluorescence angiography (**B**,**C**). Reprinted from A. Le-Nguyen et al. Indocyanine green fluorescence angiography in pediatric intestinal resections: A first prospective mixed methods clinical trial. *J Pediatr Surg* **2023**, *58*, 82–88, with permission from Elsevier [35].

Intraoperative ICG angiography is a useful adjunct in the rapid assessment of mesenteric and intestinal perfusion. Regions of weak fluorescence correlate with areas of intestinal malperfusion and have been associated with a high risk of a non-recoverable ischemic bowel injury or complications such as strictures [30]. ICG angiography can even aid in identifying regions of malperfusion that appear perfusion on visual inspection alone [35]. Strong fluorescence is linked with high probability of vascularized bowel that can be preserved (Figure 4) [35,42,43]. One prospective trial demonstrated that the use of intraoperative ICG angiography yielded a significant decrease in the amount of bowel requiring resection when compared to standard visual inspection. In that study, visualization of perfusion by ICG fluorescence changed surgeons’ operative plan in 7% of cases and favored an average of 2.5 cm more bowel preservation [35]. Once resection and/or anastomosis is complete, ICG is also efficacious in confirming adequate perfusion to anastomoses prior to case termination [7,152].

Beyond assessment of perfusion, ICG angiography is aids in anatomic delineation during minimally invasive operations for more complex pathologies such as duodenal atresia or malrotation. ICG angiography may aid in identifying and preserving vessels hidden within fibrous bands during laparoscopic Ladd procedures that would otherwise be palpable during an open approach [153]. Additionally, if ICG is administered enterally via nasogastric tube, there can be a sharp demarcation of fluorescence at the location of a duodenal web in cases of duodenal atresia [50,127].

#### 4.2.3. Colorectal

Akin to small bowel perfusion, ICG angiography facilitates intraoperative evaluation of colorectal perfusion. The variability of the pediatric marginal artery diameter has been cited as one of the factors impacting anastomotic integrity in colorectal cases [88,93]. In Hirschsprung disease, rates of complications such as strictures or leaks can present in up to 19% of patients, highlighting the importance of proper vascular evaluation of colonic perfusion in these cases [88]. In one analysis, ICG-guided resection lines for Hirschsprung’s-related reconstructions were on average 10 cm more proximal than initially intended based on visual inspection alone [88,93]. ICG angiography aided not only in determining resection lines and performing colonic de-rotations, but also in verifying perfusion to J pouches, pull-throughs, and colorectal anastomoses [31,34,89,90,91,92].

In complex reconstructions of anorectal malformations, rectovesicular malformation, and cloacal malformations, ICG angiography aids in anatomic delineation as well as perfusion assessment. In cases of rectovesicular fistulae, ICG instillation into the bladder facilitates rapid identification of the fistula site by fluorescent guidance, allowing for targeted dissection and fistula repair [34]. In anorectal malformations, ICG angiography aided surgeons by defining the location of proximal resection and delineating the distal end of the rectum that required resection due to poor fluorescence [50,154]. One study of cloacal reconstructions demonstrated that ICG angiography directly guided the operative plan in 1/3 of cases of cloaca where bowel visually appeared viable initially but upon ICG angiography demonstrated reduced perfusion, resulting in additional bowel resection to a well-perfused margin [93]. During sagittal anorecovaginourethroplasty and other perineal repair/reconstruction, ICG angiography has also verified perfusion to these delicate reconstructions [31]. ICG angiography may help reduce anastomotic and reconstructive complications postoperatively by identifying bowel segments with optimal perfusion and directing resection of malperfused bowel segments.

### 4.3. Non-Hepatobiliary Oncology

#### 4.3.1. Lymph Node Identification

**Figure 5:**
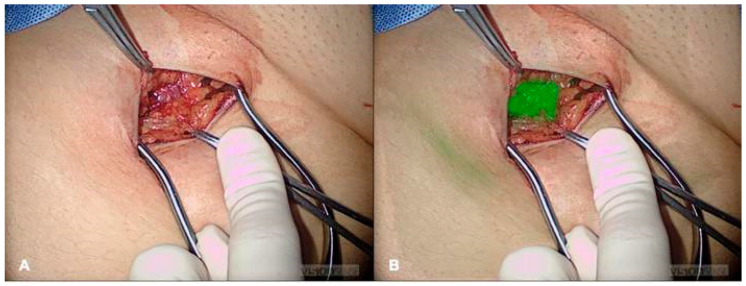
Subcutaneous intraoperative findings of the sentinel lymph node area without (**A**) and with indocyanine green detection (**B**). Reprinted from L. Pio et al. Sentinel lymph node mapping with Indocyanine green fluorescence (ICG) for pediatric and adolescent tumors: a prospective observational study. *Sci Rep* **2024**, *14*, 30135. Figure is licensed under Creative Commons-NC-ND License [105].

Sentinel lymph node biopsy or formal lymphadenectomy is not indicated in many pediatric malignancies relative to the adult oncology cohort [155]. Identification of lymphatic vessels and lymph nodes can prove challenging, often warranting preoperative lymphoscintigraphy with Tc-99 in many cases and intraoperative triangulation with a gamma probe or use of blue dyes injected subcutaneously. ICG lymphangiography has been proposed as an adjunct or even alternative approach to lymph node identification in the pediatric population (Figure 5). When ICG is injected subcutaneously for lymphangiography, lymphatic vessels and nodes that drain a tumor of interest will fluoresce, often transcutaneously in the pediatric population [103]. In younger patients, ICG may also be fluorescent directly through the skin in up to 80% of the population [106]. ICG fluorescence is noted in up to 100% of Tc-99 radioactive nodes [103,104,105]. One prospective study of numerous soft tissue malignancies (sarcoma, squamous cell carcinoma, and melanoma) found ICG fluorescence had an associated sensitivity of 84% for detecting pathologically positive lymph nodes and was non-inferior to Tc-99 guided localization of sentinel lymph nodes [106]. When compared to isosulfan blue, ICG fluorescence was found to have a higher sensitivity for identifying sentinel lymph nodes. However, it was associated with reduced specificity, and therefore a higher false positivity rate [106]. The risk of isosulfan blue dye injection—including a higher risk of anaphylaxis, skin and soft tissue necrosis, hemodynamic instability, and discoloration of the surgical bed—should be weighed against the risk of additional lymph node biopsy when considering which of these two dyes to use [104]. Combinations of these various methods can be implemented to aid in the identification of sentinel lymph nodes, although the sensitivities, specificities, and risks of each should be considered.

Within urologic oncology, intra-lesional injection of ICG also aids in the assessment of lymphatic drainage and resection of sentinel nodes. Direct injection of ICG into the renal parenchyma or the hilum allows for visualization of draining lymphatics and sentinel nodes [97,107]. For all patients, there was an increase in lymph node yield following ICG injection, and no other complications associated with the ICG injection [156]. For pediatric paratesticular rhabdomyosarcoma, retroperitoneal lymph node dissection is the standard approach but has significant perioperative morbidity [101]. An alternative approach that has been proposed is ICG injection into the spermatic cord to intraoperatively identify draining lymph nodes for targeted harvesting [157]. Pathologic evaluation of these harvested nodes from 4 patients showed no false negative or false positive lymph nodes (16/16 truly pathologic). These findings underscore the possible impact on the surgical approach offered by intralesional ICG injection.

#### 4.3.2. Pancreatic

While uncommon in the pediatric population, there are numerous pancreatic masses requiring targeted enucleation or formal resection [158]. Due to difficulty identifying some of these lesions intraoperatively, ICG angiography has been implemented to aid in targeting the lesions. In cases of hyperinsulinemia or insulinoma, pathologic nodules have been noted to fluoresce relative to the remainder of the pancreas [50,71,73]. Targeted resection, especially in cases of multifocal nodules, improves the chance of preserving pancreatic function, highlighting how ICG can improve postoperative outcomes in this population. In other pathologies, such as solid pseudopapillary neoplasm of the pancreas and persistent hyperinsulinemia with hypoglycemia, ICG angiography did not aid in lesion identification due to total fluorescence of the pancreas [73].

#### 4.3.3. Adrenal/Neuroendocrine

Adrenal tumors, including pheochromocytoma, neuroblastoma, and primary adrenocortical carcinoma, collectively have variable ICG fluorescence success intraoperatively [20,73,74]. Surgeons have successfully used ICG fluorescence to direct resection of para-aortic paraganglioma, and the fluorescence pattern even guided surgeons to resect a second foci that was ultimately pathologically positive [159]. Similarly to findings in hepatoblastoma, neuroblastomas that underwent neoadjuvant chemotherapy demonstrated reduced proliferation and were not fluorescent intraoperatively [74]. While ICG angiography may not be as efficacious for lesion delineation in adrenal and neuroendocrine pathologies, it is useful in identifying the vascular supply to these hyper-vascular tumors which can result in significant bleeding if not properly controlled [101].

### 4.4. Cardiothoracic

#### 4.4.1. Pulmonary Metastases

**Figure 6:**
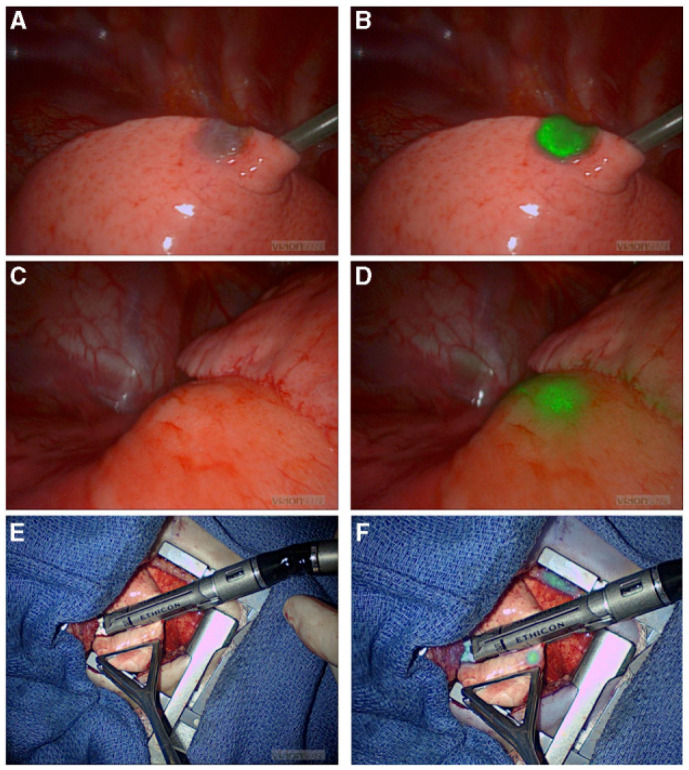
NIR-guided localization of pulmonary metastases for three thoracoscopic resections from a hepatic primary tumor. (**A**,**B**) NIR localization of a superficial nodule. This nodule was seen with both standard of care white light (**A**) and NIR (**B**). NIR, near-infrared. (**C**,**D**) NIR localization of a 2 cm nodule seen on the preoperative CT scan, but not visible when seen by standard of care white light (**C**). (**D**) The same deep nodule was localized with NIR. NIR, near-infrared; CT, computed tomography. (**E**,**F**) A small 0.2 cm nodule not localized with preoperative CT scan or with standard of care white light/tactile feedback (**E**). (**F**) The same nodule localized by NIR. NIR, near-infrared. Reprinted from A. Abdelhafeez et al. Indocyanine Green-Guided Pediatric Tumor Resection: Approach, Utility, and Challenges. *Front Pediatr* **2021**, *9*, 689612. Figure is licensed under Creative Commons-BY License [20].

Within pediatric cardiothoracic surgery, ICG is most commonly implemented during targeted resection of pulmonary metastases. In patients with pulmonary metastases from hepatoblastoma, sarcomas, or other malignancies, ICG has been implemented to highlight metastases and guide pulmonary metastasectomy. Preoperative intravenous injection of ICG can help successfully target pulmonary hepatoblastoma metastases for intraoperative fluorescence due to the delayed ICG clearance from the hepatic tissue present in the lung (Figure 6) [11,31,38,58,62,67,68,73,75,76]. ICG fluorescence is up to 98–100% sensitive for the identification of pathologically positive pulmonary hepatoblastoma metastases [61,68,77]. Fluorescence is even seen in micro-metastases as small as 0.062 mm [68]. The high sensitivity of this technology has facilitated resection of multitudes of fluorescent nodules that were not initially seen on preoperative imaging but were ultimately pathologically positive [20,59,61,68,69,72,78]. These minimally invasive approaches are classically associated with the drawback of not allowing palpation by the surgeon for metastatic disease in open thoracotomy. On pathologic examination, fluorescent margins appear congruent with histologic margins [61]. However, surgeons should be aware that lesions > 10 mm from the lung’s surface may not adequately fluoresce intraoperatively and may initially be missed unless manually compressed, in which case fluorescence may appear through the compressed parenchyma [73]. As hepatoblastoma metastases are most commonly on the periphery of the lung parenchyma, ICG fluorescence is successful in the majority of cases [75]. While ICG fluorescence is highly sensitive for pulmonary hepatoblastoma metastases, specificity is cited to be as low as 50%, leading to numerous pathologically negative resections [19,20,61,68,73,77]. Falsely fluorescent pulmonary nodules have been linked to hemorrhage, inflammation, vascular irregularity, granuloma, necrosis, or even Vicryl suture material [61,77,160].

Surgeons have also noted alternative pathologies such as osteosarcoma and melanoma metastases to be ICG fluorescent intraoperatively, aiding in targeted resection [74]. However, not all pediatric solid malignancies that have pulmonary metastases are ICG fluorescent, with ICG failing to localize myofibroblastic tumors, Ewing sarcoma, atypical cartilaginous tumors, neuroblastomas, nephroblastoma, adrenocortical carcinomas, and papillary thyroid carcinomas [73,74,78]. To counteract this effect, image-guided intra-lesional ICG injection into metastases has been described for non-hepatic derived tumors that may have reduced fluorescence with IV administration of ICG [16,110]. CT-guided ICG + methylene blue injection into pulmonary nodules has also aided in successful intraoperative localization of small pulmonary nodules, reducing the size of segmentectomy necessary to adequately resect the pathology [160]. The utilization of ICG fluorescence for visualization of pulmonary metastatic deposits can further enable safe oncologic resection outcomes in minimally invasive approaches to pulmonary metastasectomy. Surgeons performing pulmonary metastasectomy should tailor ICG use to patient pathology and be aware of the high sensitivity yet low specificity for hepatoblastoma metastatic disease burden.

#### 4.4.2. Congenital Lung Malformations

**Figure 7:**
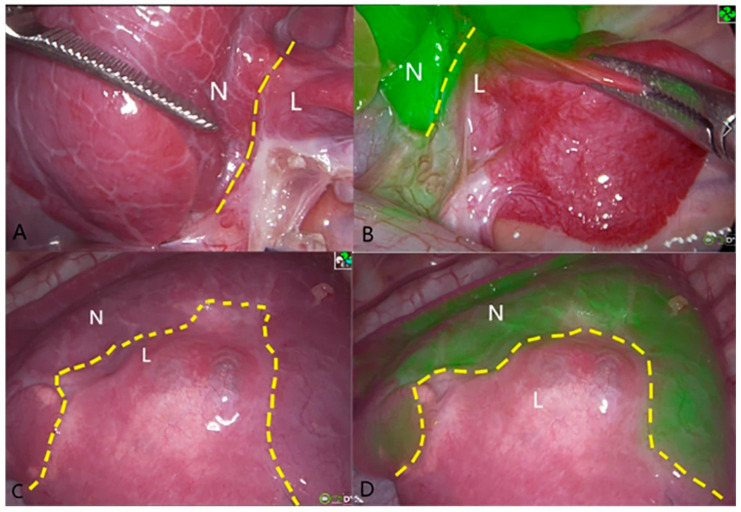
(**A**,**B**) displayed the existing lesion demarcation (yellow dotted line) and fluorescent one delineated by NIR imaging on eBPS patient in which the lesion joins with the normal lung (case 1), respectively; (**C**,**D**) shown lesion demarcation identified by naked eyes and fluorescent one in iBPS patient, respectively. (eBPS, extralobar bronchopulmonary sequestration; iBPS, intralobar bronchopulmonary sequestration; CPAM, congenital pulmonary airway malformation; NIR, near-infrared; N, normal; L, lesion). Reprinted from T. He et al. Fluorescence imaging-assisted thoracoscopic anatomical lesion resection in treating congenital lung malformation. *Sci Rep* **2025**, *15*, 755. Figure is licensed under Creative Commons-NC-ND License [83].

Congenital pulmonary airway malformation (CPAM) and pulmonary sequestration are classically managed via anatomic lobectomy, but non-anatomic resections are an alternative approach to these pathologies. ICG-guided, lesion-specific segmentectomy offers an improved parenchymal sparing option for these patients due to the superior anatomic delineation [16,81,161]. ICG angiography allows surgeons to rapidly identify the arterial supply to the diseased pulmonary segment for easier ligation prior to segmentectomy [81]. Even in pathologies requiring lobectomy, such as bronchial atresia, ICG angiography aids in vessel identification and ligation intraoperatively [82]. The fluorescence pattern will also demarcate the boundary of healthy parenchyma, directing lines of resection [80]. Alternatively, transbronchial or inhaled injection of ICG can be used to highlight the diseased parenchyma to guide resection borders. Inhaled ICG incurs green fluorescence in healthy parenchyma, whereas pathologic tissue does not fluoresce, which can further demarcate resection lines (Figure 7) [83,129]. For intra-lobar pulmonary sequestration, the use of inhaled ICG was associated with reduced time to chest tube removal and decreased time to discharge when compared to standard approaches [128]. Furthermore, postoperative ventilatory function was improved in the ICG group [128].

#### 4.4.3. Congenital Cardiac Defects

In congenital cardiac surgery, ICG angiography has proved to be a useful adjunct in assessing delicate reconstructions and anatomy. ICG angiography has been successfully implemented to determine intraoperative patency of Blalock-Taussig shunts, coronary reimplantation, and coarctation repairs [45,46]. Improved assessment of these reconstructions may facilitate immediate revision if necessary, rather than delayed diagnosis and revision. ICG angiography in these cases also aids in visualization of the small infant coronary anatomy, allowing surgeons to better avoid injury to these critical structures [39,46]. In cases of aorto-coronary fistula, ICG angiography has aided in the localization of fistula implantation and minimally invasive ligation of the fistula [44]. ICG angiography is not universally efficacious in congenital cardiac surgery, with reconstructions such as Fontan and pulmonary artery reconstruction not demonstrating adequate fluorescence [45,46].

### 4.5. Urology

#### 4.5.1. Varicocele

**Figure 8:**
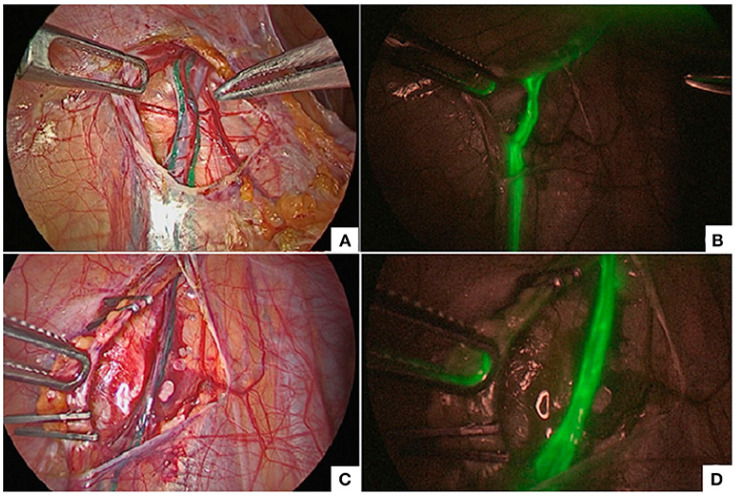
Lymphatics sparing at standard white light (**A**) and ICG-guided NIRF (**B**). Clipping and division of the spermatic bundle at standard white light (**C**) and ICG-guided NIRF (**D**). Reprinted from C. Esposito et al. Image-Guided Pediatric Surgery Using Indocyanine Green (ICG) Fluorescence in Laparoscopic and Robotic Surgery. *Front Pediatr* **2020**, *8*, 314. Figure is licensed under Creative Commons-BY License [48].

Varicocelectomy involves ligation of spermatic veins either by an open subinguinal technique or a laparoscopic technique. The laparoscopic Palomo technique is prevalent in pediatric urology, and the lymphatic sparing modification to the technique is associated with reduced risk of post-operative hydrocele [16]. When ICG is injected intra-testicularly, lymphatics within the spermatic cord can be thoroughly visualized and spared during the procedure (Figure 8) [16,34,48,73,99,130,162]. Successful visualization of lymphatic vessels is seen in up to 100% of cases [99,162]. Furthermore, ICG angiography during varicocelectomy aids in visualization of the testicular vein, although if the vessels spasm intraoperatively due to manipulation sensitivity may suffer [34,44,163,164,165]. Use of ICG lymphangiography for varicocelectomy has been linked to a reduced risk of postoperative hydrocele and recurrence [44,54,162,166].

#### 4.5.2. Testicular Torsion

The traditional methods for determining testicular viability include visual examination of testicular blood flow following detorsion or the presence of bleeding after incising the testicular parenchyma. Intraoperative ICG angiography has supplemented these methods, with some using a fluorescence cutoff of 5 min to determine whether to proceed to incision of the parenchyma [167,168]. If the testis was not fluorescent, then it was incised to evaluate for bleeding. If bleeding was present, orchiopexy was performed; otherwise, orchiectomy was indicated due to malperfusion. One case series demonstrated ICG fluorescence to black appearing testis that would have likely otherwise undergone orchiectomy, directly guiding the surgeons towards testicular salvage [169]. These patients had preserved testicular flow and no signs of testicular atrophy on follow up [169]. ICG angiography is efficacious in determining perfusion to torsed testes and may improve rates of testicular salvage in the pediatric population.

#### 4.5.3. Nephrectomy

During nephrectomy for both benign and malignant processes, intraoperative ICG angiography improved visualization of variable renal vasculature, ensuring safe dissection and ligation of vessels [34,38,44,48,99,140]. As mentioned previously, the timing of ICG injection can impact whether a tumor or the healthy renal parenchyma is the fluorescent target. Intraoperative injection will yield fluorescence of the healthy renal parenchyma. The high fluorescence of the renal parenchyma has negatively impacted the delineation of tumor margins on ICG angiography in prior studies. In Wilms tumors, there is high background noise, resulting in difficulty determining true margins [140]. Beyond identifying the primary tumor and its vasculature, ICG has been utilized for lymph node identification during minimally invasive radical or partial nephrectomy for renal tumors. Direct injection of ICG into the renal parenchyma or the hilum to allow for visualization of draining lymphatics and sentinel nodes [97]. For all patients, there was an increase in lymph node yield following ICG injection, and no other complications associated with the ICG injection [156].

#### 4.5.4. Hemi-Nephrectomy

A duplex kidney is a congenital malformation that afflicts approximately 1% of the population [170]. Due to the risk of urinary stasis, reflux, or obstruction, patients are at risk for infectious complications and nephropathy. Hemi-nephrectomy for this pathology is directed at the resection of the non-functional moiety and leaving the functional renal parenchyma in situ. ICG angiography aids in delineating the anatomic border between functional and non-functional parenchyma [16,31,73,99,171,172]. ICG angiography can also serve as an early warning of aberrant anatomy to reduce the risk of inadvertent injury to the functional moiety [171]. ICG angiography during hemi-nephrectomy was associated with a faster operation with fewer postoperative complications [99]. ICG helps identify crossing vessels or aberrant vascular anatomy [14].

During hemi-nephrectomy for nephroblastoma, ICG guidance had equivalent margin rates; however, the healthy parenchyma glows brighter than nephroblastoma, which is different than other pathologies listed here [17,97]. In Wilms tumor and renal cell carcinoma, there is adequate delineation of the border between the tumor and the healthy parenchyma. This fluorescent delineation can be clarified by clearing any peri-nephric fat prior to ICG angiography [98]. ICG may also facilitate minimally invasive resection of tumors that otherwise are typically resected via an open approach (>6 cm) due to improved visualization of feeding vessels [101]. If the ICG is injected 24 h preoperatively, the tumor will fluoresce, and the healthy parenchyma will not [73]. ICG fluorescence is not universally helpful in the resection of renal malignancies, with one case of a malignant rhabdoid tumor of the kidney having an unclear fluorescent border that could not be directly followed [59].

#### 4.5.5. Congenital Malformations

ICG angiography is also efficacious in determining perfusion to delicate reconstructions of hypospadias repair and bladder exstrophy reconstructions. Onlay preputial island flap has been a longstanding method for managing hypospadias [173,174]. Its complications have mostly been related to issues with tissue perfusion, which historically could only be evaluated with visual techniques. ICG angiography is now being used to assess the perfusion of the flap as well as its pedicle in efforts to minimize the complications of tissue malperfusion, such as wound dehiscence or stricture [173]. There is debate regarding the most appropriate method for bladder exstrophy reconstruction, with the one-stage delayed bladder closure and radical soft tissue mobilization being plagued by complications of poor tissue perfusion and possible penile loss [175,176]. ICG angiography throughout reconstruction to ensure appropriate tissue perfusion can verify penile perfusion, establishing ICG’s use as a potential adjunct to assess tissue perfusion in these complex reconstructions [176].

#### 4.5.6. Ureteral Identification/Reconstruction

Placement of ureteral stents has typically been used to aid in intraoperative identification of the ureters during both open and laparoscopic surgery. As an alternative approach that is easily viewed minimally invasively, ICG was able to be instilled by directly cannulating the ureter with ureteroscopy and allowed for easy identification of the ureters without the need for stent placement [177]. In addition, during ureteral reconstruction following ureterotomy, ICG was used to confirm adequate ureteral perfusion [178].

#### 4.5.7. Cyst De-Roofing

In the de-roofing of simple renal cysts, ICG fluorescence identified the avascular cystic dome, promoting safer fenestration in several cases [48,99,100]. Intravenous ICG has been used during renal cyst unroofing to delineate normal renal parenchyma versus non-fluorescent renal cyst. There were no differences in operative time or success rate with the use of ICG.

### 4.6. Lymphatic Pathologies

#### 4.6.1. Chylothorax

Chylothoraxes can occur congenitally, postoperatively, or secondary to trauma. While many can be managed conservatively, high volume or persistent chyle leaks often require surgical intervention. ICG lymphangiography via subcutaneous injection in the interdigital space of the first and second toes or the inguinal regions facilitates easier identification of the thoracic duct and the exact source of chyle leak within the thorax due to the leakage of fluorescent dye. This precise localization of chyle extravasation allows for a more accurate dissection of the thoracic duct and ligation/repair of lymphatic vessels [7,34,38,114,117,118]. ICG-guided thoracoscopic lymphatic leak ligation has proved up to 90% successful in cases of medically refractory chylothoraxes [17,115]. In cases where surgeons cannot identify the site of chyle leak despite ICG lymphangiography, ligation of the thoracic duct may be required [179]. Even preoperatively, ICG can be injected into extremities subcutaneously at the bedside in the intensive care unit, and fluorescence patterns can aid in determining the site of lymphatic leakage and ultimately direct the surgical plan [116].

#### 4.6.2. Chylous Ascites

In neonates with idiopathic or traumatic lymphatic ascites, ICG lymphangiography has also been used to analyze lymphatic flow and dysfunction, guiding procedural/surgical management akin to cases of chylothorax [34,112,113]. Alternatively, ICG may be injected into lower extremity lymphatic vessels under CT guidance to identify sites of chyle leak intra-abdominally, guiding laparoscopic ligation of the lymphatics [40]. There are limited case reports of ICG not aiding in the localization of lymphatic leaks, and most reports report significant clinical assistance [50,116].

#### 4.6.3. Lymphedema

In pediatric patients with primary lymphedema, ICG lymphangiography can be used to evaluate superficial lymphatics transcutaneously, while Tc-99 can be used to characterize the deep lymphatics that are not fluorescent transcutaneously [120]. In some cases, ICG may identify normal lymphatics but reduced flow, which is hypothesized to be secondary to reduced contractility of the vessels. ICG lymphangiography may therefore be able to identify patients more likely to respond to conservative therapy with compression and manual drainage [121]. Select patients may ultimately require lymphovenous bypass or vascularized lymph node transfer to treat this pathology. By properly visualizing the lymphatic flow, ICG lymphangiography can also guide the surgeon where to make the skin incision and the respective lymphovenous anastomosis [122]. Intraoperatively, the patency of these anastomoses and lymphatic flow can be confirmed with ICG fluorescence [120]. Intraoperative confirmation of these anastomoses is critical due to the high rate of obstruction in these delicate anastomoses and vessels [180].

#### 4.6.4. Lymphangioma/Lymphatic Malformations

As an adjunct to traditional imaging with ultrasonography and MRI, ICG lymphangiography can be used intraoperatively to identify malformed lymphatics in cases of pediatric lymphangioma [111,181]. This ICG-guided technique aims to exclusively excise lymphatics and tissues associated with the lymphangioma, reducing the impact on native lymphatics and the risk of postoperative lymphedema [123]. It can also guide incision location and size in cosmetically sensitive regions, such as peri-orbital regions [126].

In addition to providing intraoperative anatomic delineation, ICG lymphangiography is a useful diagnostic tool for classifying pediatric lymphatic dysplasia. By identifying retrograde lymphatic flow, ICG lymphangiography has adequately predicted clinical disease course in these patients [25,182]. The flow of ICG lymphangiography has been classified into 4 subtypes (1—strong detectable inflow, 2—small observable inflow, 3—superficial lymph flow over the lesion, and 4—flow around the lymphatic malformation without connection), which can be used to stratify treatment approaches for pediatric patients with inadequate lymphatic drainage [124].

### 4.7. Gynecology

#### 4.7.1. Ovarian Perfusion

In cases of spontaneous ovarian torsion or ovarian torsion secondary to a cyst or mass, ICG angiography can be used to assess the viability of ovarian perfusion after it is detorsed [34]. Not only does the angiography aid in the removal of cysts and masses from the healthy ovarian tissue, but ICG angiography is associated with a reduced rate of oophorectomy in cases of torsion due to the improved perfusion assessment intraoperatively [48,183].

#### 4.7.2. Mayer-Rokitansky-Küster-Hauser Syndrome

Approaches to the management of Mayer-Rokitansky-Küster-Hauser Syndrome are variable, but one method of vaginal reconstruction is with a well-vascularized segment of bowel [184,185]. Minimally invasive approaches have been described, with laparoscopic dissection of the sigmoid colon and in the pelvis. Adequate mobilization of the colon while maintaining the patency of the vascular pedicle without twisting is imperative in these cases. ICG angiography aids in mobilization of the selected colon segment and confirms perfusion of the reconstruction internally via laparoscopy and at the perineum after reconstruction [34,186].

### 4.8. Spleen

**Figure 9:**
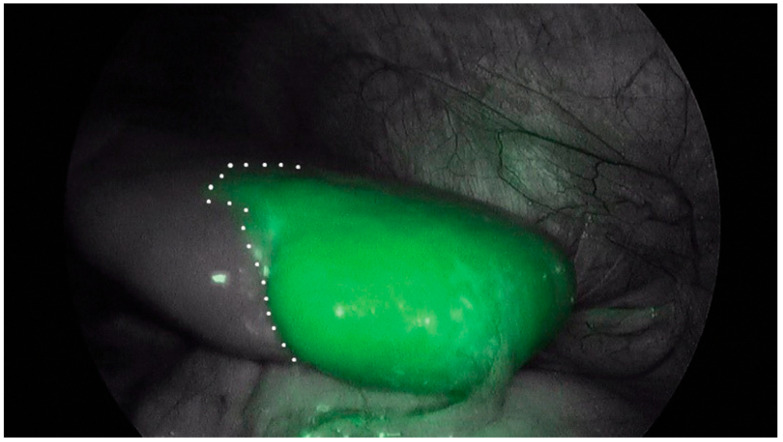
Intraoperative image after arterial clamping. Image under near-infrared light. To the right of the dotted line, inferolateral pole of the spleen. It shows fluorescence since arterial vascularization in this portion is preserved. To the left of the dotted line, supero-medial pole. It does not show fluorescence as the superior splenic artery, which supplies arterial irrigation to this portion, has been clamped. Reprinted from I. Bada-Bosch et al. Laparoscopic Partial Splenectomy Assisted by Fluorescence in a 13-Year-Old Girl. *European J Pediatr Surg Rep* **2020**, *8*, e81–e85. Figure is licensed under Creative Commons-BY License [94].

A variety of pediatric hematologic diseases ultimately require splenectomy, including thalassemias, spherocytosis, sickle cell disease, and more. This historically open procedure has been supplanted by minimally invasive splenectomy becoming more prevalent. ICG angiography has been implemented in the minimally invasive approach to splenectomy to more safely and rapidly dissect and ligate splenic vessels under fluorescent guidance [95]. In other benign splenic pathologies, such as splenic cysts, requiring partial splenectomy, ICG angiography also aids in visualization of hilar vessels and transection planes. Adequate identification of vessels supplying the pole of the spleen prior to partial splenectomy allows for a safer dissection in this hyper-vascular organ [94,95,96]. Once vessels are ligated, the plane of transection is delineated by the fluorescence and the remnant’s perfusion can also be confirmed before case termination (Figure 9).

### 4.9. Soft Tissue/Flap Perfusion

Numerous pathologies may require tissue coverage, including tissue loss after trauma or oncologic resection. Viability of skin after trauma is historically assessed visually alone, and surgeon judgment is what determines resection of de-vascularized segments. ICG angiography augments this assessment by demarcating areas of perfusion in fluorescent green and regions of malperfusion being less or non-fluorescent. There are ongoing efforts to quantify fluorescence using post-operative vascular imaging software to determine segments at risk for necrosis post-operatively [37]. If coverage is not feasible primarily, myocutaneous flaps are an option for definitive coverage, but are not without risks due to the supermicrosurgical techniques required. In the pediatric population, small vessel size and superficial distribution places local and free flaps at significant risk of technical error at index operation requiring urgent return to the operating room [187]. ICG angiography allows surgeons to verify patency of anastomoses intraoperatively and assess flap perfusion [187,188,189]. In patients requiring autologous ear reconstruction, use of ICG angiography was associated with reduced risk of requiring surgical revision, likely due to improved assessment of flap perfusion [33]. ICG lymphangiography can also be used postoperatively to assess for lymphatic congestion within a flap and visualize lymphatic connections across a flap margin [190]. Similarly to the assessment of neonatal coronary vessels, ICG angiography is particularly useful in the assessment of flap microvasculature intraoperatively.

### 4.10. Otolaryngology

Head and neck malignancies represent up to 12% of all pediatric malignancies [102]. Preoperative administration of ICG intravenously facilitates selective fluorescence of these malignancies. If administered locally intraoperatively, ICG fluorescence can aid in anatomic delineation during resection of benign thyroglossal, branchial cleft cysts, and nasal cysts [111,181]. ICG fluorescence is cited to have a sensitivity of 83% and specificity of 88% for head and neck malignancies [102]. Intraoperative fluorescence patterns have directly prompted changes in operative planning in head and neck malignancies, with one case reporting a conversion to bilateral neck dissections based on fluorescence patterns, which were consistent with local metastases [102]. Regrettably, pathologic concordance with fluorescence pattern is not as strong in head and neck malignancies when compared to hepatoblastoma. There were pathologically positive margins without fluorescence in up to 25% of cases of head and neck malignancies [102,140]. Further investigation into the efficacy of ICG for head and neck malignancies is required.

### 4.11. Ophthalmology

ICG fluorescence has also been shown to be a valuable adjunct in ophthalmology. In their case series, Keren et al. described the use of ICG to improve visualization of the cyst capsule during surgical excision of orbital lacrimal duct cysts after inadvertent cyst rupture [191]. Of the six patients included in their study, none had evidence of cyst recurrence during follow-up or naso-lacrimal duct obstruction.

Another area where ICG may also hold promise is in cataract surgery during creation of a continuous curvilinear capsulorrhexis (CCC), where adequate visualization of the capsule flap is desired [192]. Some authors have reported that ICG is effective in not only staining the capsule, but also allowing for easier visualization compared to other dyes [193].

### 4.12. Neurosurgery

ICG angiography is also used in the intraoperative assessment of several neurosurgical conditions [7]. Moya Moya disease is a disorder characterized by stenosis of the internal carotid artery and is associated with the formation of collateral vessels, also known as Moya Moya vessels [194]. Multiple authors have utilized ICG angiography to evaluate the patency of superficial temporal artery-middle cerebral artery bypass in the treatment of Moya-Moya disease [195,196]. Other studies have used ICG angiography to explore the relationship between bypass flow and the development of postoperative hyperperfusion [197,198]. ICG angiography has also shown promise in the assessment of pediatric aneurysms and arteriovenous fistulas. For instance, some authors have commented on its utility in evaluating blood flow dynamics and aneurysmal inflow and outflow during aneurysmectomy [199,200]. In complex aneurysms requiring bypass, others have used ICG angiography to evaluate bypass patency [201]. The fluorescence pattern has also successfully identified and directed ligation of a pial arteriovenous fistula [202]. ICG angiography can also be used to determine the perfusion of skin flaps after craniosynostosis reconstruction, as these flaps may be under significant tension after skull remodeling [32]. Extracranial neurosurgical pathologies may also be assessed with ICG angiography. During myelomeningocele repair, ICG angiography delineated viable neural tissue in a newborn with myelomeningocele [203].

## 5. Conclusions and Future Directions

ICG is a useful adjunct in the pediatric surgeon’s armamentarium. Like any technology, there remain limitations to ICG, and its use cases should be strongly considered preoperatively. At the current time, ICG fluorescence is largely a qualitative measurement rather than quantitative, and objective cutoffs for adequate perfusion have not been established. Ongoing efforts should aim to quantify the speed and strength of fluorescence to have standardization in what is deemed adequate perfusion or malperfusion. Furthermore, within pediatric oncology, there remain falsely positive and negative fluorescent lesions depending on specific neoplastic pathologies. Further longitudinal evaluation of ICG fluorescence in a variety of pathologies is still required to adequately determine how to best implement this technology. For pathologies with more supporting data, such as hepatoblastoma, future analyses should evaluate rates of histologically negative margins in operations with ICG fluorescence compared to those without. Finally, hospital system-wide value-based analysis is still required in the pediatric population, as there may still be system-based limitations to the implementation of ICG in some centers. The pediatric surgical applications of ICG demonstrate extreme promise in augmenting surgical efficacy, efficiency, and reducing patient morbidity. Future analysis should continue to address best practices within the pediatric population regarding its implementation to reduce overall healthcare costs and optimize patient outcomes.

## Figures and Tables

**Table 1:** Inclusion and Exclusion Criteria.

Inclusion Criteria	Exclusion Criteria
Patient population ≤18 years old	Patient population >18 years old
Use of ICG for preoperative planning or diagnostics	Use of alternative fluorescent dye (e.g., fluorescein)
Use of ICG for intraoperative guidance or diagnostics	Full manuscript not available in English
Use of ICG for postoperative diagnostics or prognostication	

## Abbreviations

The following abbreviations are used in this manuscript:

ICG	Indocyanine Green
NIR	Near-Infra Red
IOC	Intraoperative Cholangiography
CPAM	Congenital Pulmonary Airway Malformation
